# The relationship between different exercise modes and visuospatial working memory in older adults: a cross-sectional study

**DOI:** 10.7717/peerj.2254

**Published:** 2016-07-20

**Authors:** Wei Guo, Biye Wang, Yue Lu, Qin Zhu, Zhihao Shi, Jie Ren

**Affiliations:** 1 School of Kinesiology, Shanghai University of Sport, Shanghai, China; 2 Division of Kinesiology and Health, University of Wyoming, Laramie, WY, United States of America; 3 China Table Tennis College, Shanghai University of Sport, Shanghai, China

**Keywords:** Visuospatial working memory, Exercise modes, Older adults

## Abstract

The purpose of the study was to investigate the relationship between different exercise modes and visuospatial working memory in healthy older adults. A cross-sectional design was adopted. A total of 111 healthy older adults were enrolled in the study. They were classified by the exercise-related questionnaire to be in an open-skill group, closed-skill group or sedentary group. In experiment 1, the participants performed a visuospatial working memory task. The results indicated that both closed-skill (*p* < 0.05) and open-skill (*p* < 0.01) groups reached a higher accuracy than the sedentary group. Experiment 2 examined whether the exercise-induced benefit of working memory was manifested in passive maintenance or active manipulation of working memory which was assessed by visuospatial short-term memory task and visuospatial mental rotation task, respectively. The results showed that the open-skill (*p* < 0.01) group was more accurate than the sedentary group in the visuospatial short-term memory task, whereas the group difference in the visuospatial mental rotation task was not significant. These findings combined to suggest that physical exercise was associated with better visuospatial working memory in older adults. Furthermore, open-skill exercises that demand higher cognitive processing showed selective benefit for passive maintenance of working memory.

## Introduction

Cognitive aging is generally considered to be associated with performance decline in a multitude of cognitive tasks that require a variety of perceptual and cognitive processes. Working memory, the ability to maintain and consciously manipulate information, is particularly age-sensitive (Baddeley et al., 1999; Kirova, Bays & Lagalwar, 2015). Working memory consists of a central executive system and two independent slave sub-systems: the phonological loop and the visuospatial sketchpad (Baddeley, 1998). Aging-related decline of working memory has been found in visuospatial span tasks (Beigneux, Plaie & Isingrini, 2007), verbal span tasks (Vecchi, Richardson & Cavallini, 2005), and multiple executive functions tasks (Piolino & Cmartinelli, 2010). Previous studies have indicated that working memory reacts sensitively to environmental changes and can be significantly improved after cognitive training (Blacker et al., 2014; Carretti, Borella & De Beni, 2007). Similarly, several studies have also revealed that working memory may benefit from physical exercise or aerobic fitness (Chang et al., 2013; Padilla, Pérez & Andrés, 2014). However, the beneficial effect on working memory did not show in some fitness-cognition studies. Kamijo et al. (2010) used maximum oxygen consumption (VO_2_max) to categorize participants as higher-fit and lower-fit groups and concluded no relationship between aerobic fitness and working memory (Kamijo et al., 2010). The possible reason for the inconsistent findings may mainly due to the differences between physical exercise and fitness measures. Also, the working memory tasks used in these studies focus on the different aspect of working memory. The Sternberg task used in Chang’s and Kamijo’s study focused mainly on the maintenance component of working memory, while automatic operation span task applied in Padilla’s study measured both maintenance and manipulation. Also, young adults reach their peak of cognitive performance, which may interfere the possible association between physical exercise (fitness) and cognition (Voss et al., 2011). Therefore, it could be another possible reason for the inconsistent findings as the subject populations varied from young adults (Padilla, Pérez & Andrés, 2014; Kamijo et al., 2010) to older adults (Chang et al., 2013).

Although the mechanism underlying cognitive aging is not fully understood, several studies support the notion that physical exercise is a significant moderator for age-related cognition decline. For example, after a 1-year aerobic exercise intervention, hippocampal volume increased 2% in older adults (Erickson et al., 2011), effectively reversing the tendency for hippocampal shrinkage with aging. Although many studies have shown the effectiveness of physical exercise on preventing cognitive aging, only a few have compared the cognitive benefits received from different exercise modes (e.g. open-skill exercises vs. closed-skill exercises). These studies revealed that older adults who participated in open-skill exercise with increased cognitive demands demonstrated better executive function and inhibitory function than those who participated in the closed-skill exercise (Dai et al., 2013; Huang et al., 2014). The classification of open-skill and closed-skill exercises is based on the predictability of the performance environment. Open-skill exercises are defined as those in which exercisers are required to react in a dynamically changing, unpredictable, and externally-paced environment (e.g., table tennis and badminton). While closed-skill exercises refer to those in which the environment is relatively stable, predictable, and self-paced for exercisers (e.g., running and swimming) (McMorris, 2014). More importantly, compared to closed-skill exercises, open-skill exercises require individuals to invest higher cognitive effort in the unpredictable environment. Taking table tennis as an example, it is one of the fastest ball sports, and can be judged as a fairly difficult motor skill. Since the response window dictated by the ball speed is very brief, the player has to use advanced cues to decide what response is required (Padulo et al., 2015). Moreover, table tennis is also a highly developed tactical skill, involving creativity, concentration, competitiveness, apprehension, self-regulation, and will power (Raab, Masters & Maxwell, 2005). Additional cognitive demands in physical exercise may benefit cognition more than exercise alone (O’Dwyer, 2008). It has been shown that open-skill athletes are more flexible in visual attention, decision making, inhibition, and working memory, compared to closed-skill athletes (Voss et al., 2010; Wang et al., 2013).

The present study used a cross-sectional design to investigate the relationship between choice of exercise mode and visuospatial working memory in healthy older adults, and further examined whether the effect of choice of exercise mode was specific to passive maintenance or active manipulation of working memory. Consisting of a passive maintenance component and an active manipulation component, working memory stores information for active use and plays a role in consolidating information for long-term storage (Baddeley, 2003; Baddeley, 2012). A process-specific account of working memory, in which maintenance and manipulation are dissociable components, has been suggested (D’Esposito & Postle, 1999; Smith & Jonides, 1999). The dissociation of the two components was also proved in developmental neuroscience, for example, a study revealed that information manipulation was characterized by a longer developmental time course than information maintenance since it involved dorsolateral prefrontal and superior parietal cortex with a slower development as compared to the ventrolateral prefrontal cortex underlying maintenance (Federico, Delogu & Raffone, 2014). Previous studies explored the beneficial effects of physical exercise on working memory have focused mainly on the maintenance component (Komiyama et al., 2014; Tsai et al., 2014), while only a few have investigated the effects on the manipulation component or compared the effects on the two components. The present study took both maintenance and manipulation components into account. The passive maintenance component was tested by a short-term memory task and the active manipulation component was tested by a mental rotation task. It was hypothesized that: (1) Open-skill exercisers would have better visuospatial working memory than closed-skill exercisers and sedentary controls; and (2) open-skill exercisers would exhibit better ability on both passive maintenance and active manipulation.

## Methods

### Ethical approval

The study was carried out ethically and approved by the Ethical Committee of Shanghai University of Sport (No. 2015014).

### Experiment 1

Experiment 1 attempted to determine the differences of the visuospatial working memory among open-skill, closed-skill and sedentary groups.

#### Participants

A total of 124 participants aged between 58 and 81 years old were recruited from community senior centers. All participants were screened for dementia by the Mini-Mental Status Examination (O’Neilli, 1991) and excluded from participation if they did not reach the required cut-off score of 25. Eligible participants were additionally required to satisfy the following criteria: (1) demonstrated strong right handedness, (2) had a corrected visual acuity of at least 20/40, (3) reported to be free of psychiatric and neurological disorders, (4) had a normal body weight (body mass index (BMI) of less than 25.0 and more than 18.5) and (5) signed the written informed consent which was proved by the Ethical Committee of Shanghai University of Sport. A total of 111 participants were enrolled in the study. They were assigned to the 3 groups according to the exercise mode determined by the exercise-related questionnaires: open-skill group, closed-skill group and sedentary group. Participants were assessed with the Taiwan version of the International Physical Activity Questionnaire (IPAQ) (Liou et al., 2008) and type of exercise questionnaire. The IPAQ was used to estimate the total amount of physical activity and to classify the participants into three activity levels: inactivity, low activity and sufficient activity. The type of exercise questionnaire evaluated the specific type of exercise and the frequency per week, the duration per time and the years of participating in the specific type of exercise. Open-skill and closed-skill groups mainly participated in open-skill (i.e., table tennis) or closed-skill (i.e., jogging or swimming) exercises at least three times per week with each time for 30 min in the previous year and had to participate in these exercises for at least 1 year. Participants who took part in both type of physical exercises were excluded. The sedentary group participated in exercise at irregular base and were assessed at the inactivity or low activity level. Table 1 shows the main characteristics of the subjects in different groups.

**Table 1:** The main characteristics of the subjects in different groups.

	Sedentary (*n* = 37)	Closed-skill (*n* = 38)	Open-skill (*n* = 36)
Male	16	15	17
Age (years)	66.9 ± 5.9	66.7 ± 5.8	67.6 ± 5.9
Education (years)	11.0 ± 2.6	11.4 ± 2.9	12.6 ± 2.7
Height (cm)	162.3 ± 5.6	160.9 ± 7.0	163.7 ± 7.6
Weight (kg)	64.2 ± 10.2	62.3 ± 8.1	63.2 ± 8.7
BMI (kg/m^2^)	24.7 ± 4.1	24.0 ± 2.9	23.5 ± 2.3
MMES	27.9 ± 1.6	28.3 ± 1.3	28.2 ± 1.7
IPAQ (METs/week)	3085.3 ± 1891.9	5762.2 ± 2564.5	6086.9 ± 2162.8

**Notes.**

MMSE Mini Mental State Exam IPAQ International Physical Activity Questionnaire METs Metabolic equivalents

#### Task

**Figure 1:**
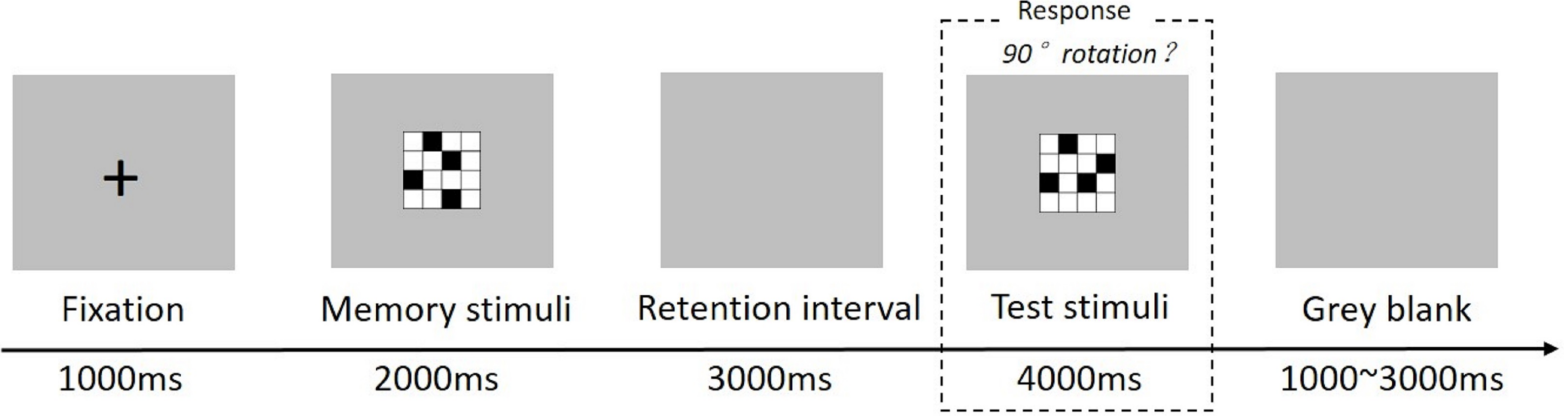
Stimuli and experimental paradigm used for visuospatial working memory.

A visuospatial working memory task (VWMT) was used for the experiment. A computer screen (19 inch) was positioned 100 cm in front of the participants. The task began with a black cross-shape fixation point (0.8° × 0.8° of visual angle) on the grey background, displayed in the center of the screen for 1,000 ms. Then, a 4 × 4 matrix (2.8° × 2.8° of visual angle) was displayed with four squares of solid black (the same stimulus used by Suchan et al. (2006) presented for 2,000 ms as a probe stimulus. Subsequently, a grey blank screen was displayed for 3,000 ms, during which subjects were instructed to mentally rotate the probe stimulus 90° to the right or the left. The testing stimulus was then presented for 4,000 ms, and subjects had to press button “1” on a numeric keyboard with their left index finger if the testing stimulus was consistent with the mentally rotated probe stimulus 90° either to the left or the right, and press button “3” with their right index finger if it was not. Once the button was pressed, the stimulus disappeared. The responses made 4,000 ms after the presentation of the test stimulus were considered as an omission error. Again a grey blank screen of 1,000–3,000 ms was randomly presented after each pair (see Fig. 1). In the experiment, 20 matching and 20 non-matching trials were presented randomly. Participants were allowed to rest every 10 trails.

#### Procedures

After arriving at the laboratory, the participants were informed of the purpose of the experiment and signed the consent form. Then the IPAQ, type of exercise questionnaire, and MMSE were completed under the instruction of the experimenter. After the participants were confirmed for eligibility for the study, they were informed about the rules of the visuospatial working memory task and received 10 practice trials with feedback. Only if their accuracy reached 70% in the practice, could they go on with the experiment. No feedback was provided during experiment.

#### Data recording and statistical analysis

Reaction time (RT, the time from the presence of the test stimuli to key press) and accuracy (AC) were collected via MATLAB, Psychtoolbox 3.0 (Kleiner et al., 2007). The RTs beyond three standard deviations of the individual mean were removed. One-way ANOVA was adopted. The group was treated as the between independent variable and the RT and AC were the dependent variables. LSD post hoc analyses were performed to further determine group differences. The significant level was set at *p* < 0.05.

#### Results

##### Participant characteristics

A one-way ANOVA revealed non-significant differences in age (F_(2,108)_ = 0.23, *p* = 0.79), education level (F_(2,108)_ = 2.97, *p* = 0.06), height (F_(2,108)_ = 1.58, *p* = 0.21), weight (F_(2,108)_ = 0.41, *p* = 0.66), BMI (F_(2,108)_ = 1.42, *p* = 0.27) and MMSE (F_(2,108)_ = 0.68, *p* = 0.51), and as expected, a significant difference was observed in physical activity level (F_(2,108)_ = 20.59, *p* < 0.01). A post hoc comparison demonstrated that the participants in the two exercise groups reached a higher level of physical exercise than the sedentary group, while no significant difference between the open and closed-skill groups existed.

##### Visuospatial working memory task results

**Figure 2:**
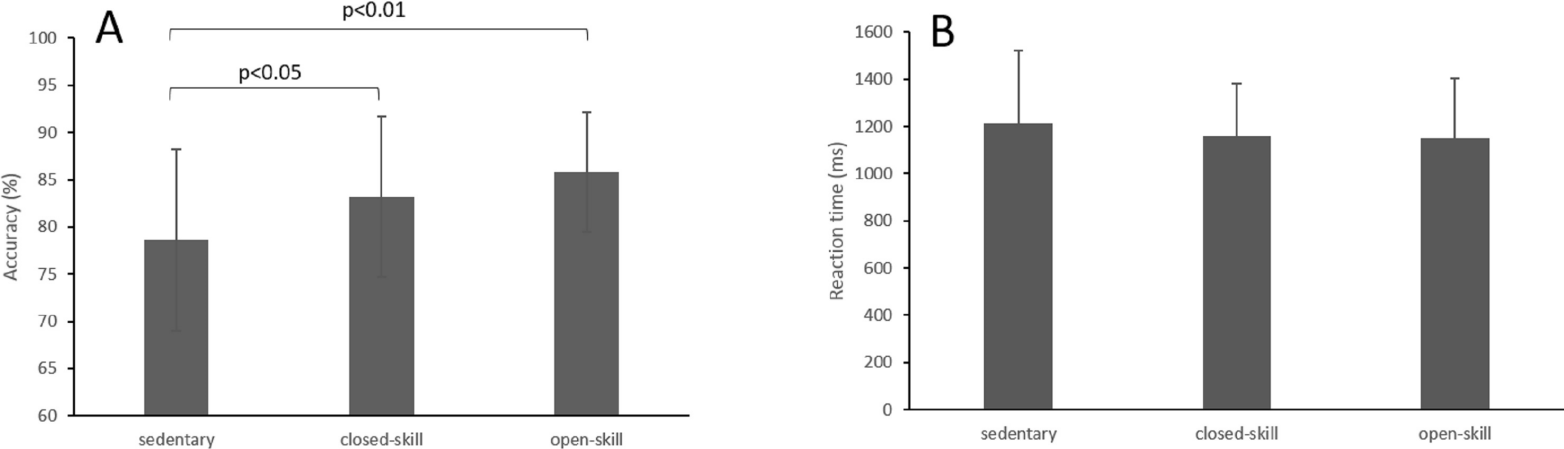
Accuracy (A) and reaction time (B) on visuospatial working memory task.

Eight participants were excluded because they could not pass the practice, and one participant was excluded due to low accuracy (40%). The number of participants in the experiment was 102 and thus each group consisted of 34 subjects. For accuracy, there was a significant difference among the groups (F_(2,99)_ = 6.71, *p* < 0.01). Post hoc analysis indicated that both closed-skill group (*p* < 0.05) and open-skill (*p* < 0.01) group reached a higher accuracy than the sedentary group, but there was no difference between the closed-skill and open-skill group. There were no differences in reaction time among the groups (F_(2,99)_ = 0.54, *p* = 0.58; see Fig. 2).

### Experiment 2

Experiment 1 confirmed better performance in the visuospatial working memory task due to participation in physical exercise. Experiment 2 was aimed to examine whether the exercise-enhanced working memory was manifested in passive maintenance or active manipulation of working memory.

#### Participants

The participants in the experiment 1 also participated in experiment 2.

#### Tasks

A visuospatial short-term memory task (VSMT) and visuospatial mental rotation task (VMTT) were adopted in the experiment. A paradigmatic example of working memory involved in active manipulation is the *n*-back task, although this task has been related to continuous updating (Wager & Smith, 2003). Active manipulation of working memory may be better achieved by other requirements such as mental rotation (Suchan et al., 2006).

**Figure 3:**
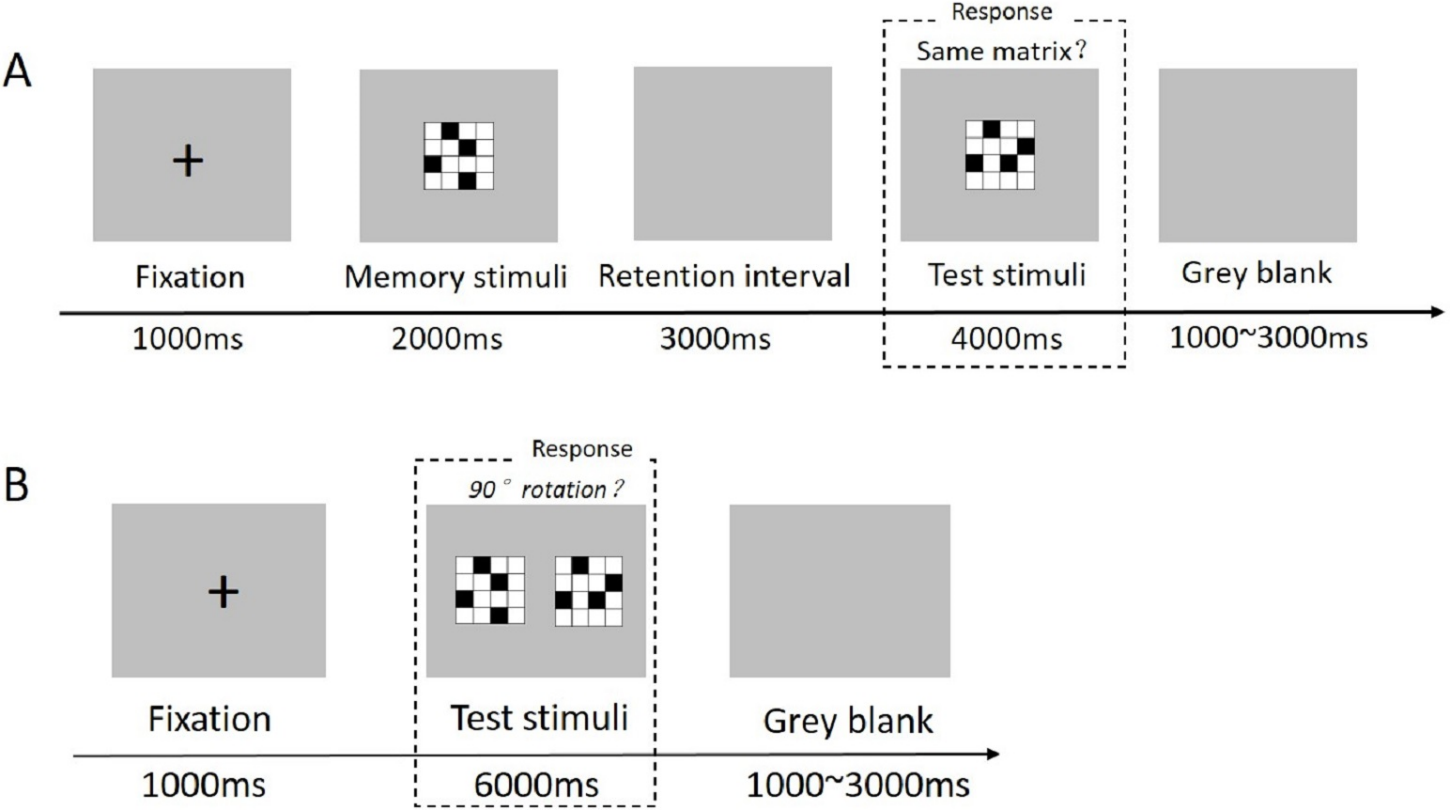
Stimuli and experimental paradigm used for visuospatial short-term memory (A) and visuospatial mental rotation task (B).

The stimulus material was the same as in experiment 1. The experimental paradigm of VSMT was similar to that of VWMT used in Experiment 1 except that subjects were instructed to retain the memory stimulus during the retention interval, and when testing stimulus was displayed, they had to determine whether or not the testing stimulus was identical to the memory stimulus by pressing a key (press button “1” with their right index finger if the testing stimulus was identical to the memory stimulus, or press button “3” if it was not; see Fig. 3). For the VMTT, the task also started with a 1,000 ms fixation point, then a pair of matrices were presented on the screen for 6,000 ms. Participants had to compare the two matrices to determine whether the matrices on the right corresponded to a 90° rotation of the matrices on the left. Inter-trial intervals were identical to the VWMT (see Fig. 3).

#### Procedures

Participants who completed experiment 1 came to the laboratory again to participate in the experiment. They were instructed about the rules of the VSMT and VMTT, received 10 practice trails with feedback, and had to reach above 70% accuracy in each. The sequence of the two tasks was counterbalanced among the participants.

#### Data recording and statistical analysis

Data recording and statistical analysis were the same as experiment 1. The one-way ANOVA was conducted for VSMT and VMTT separately.

#### Results

**Figure 4:**
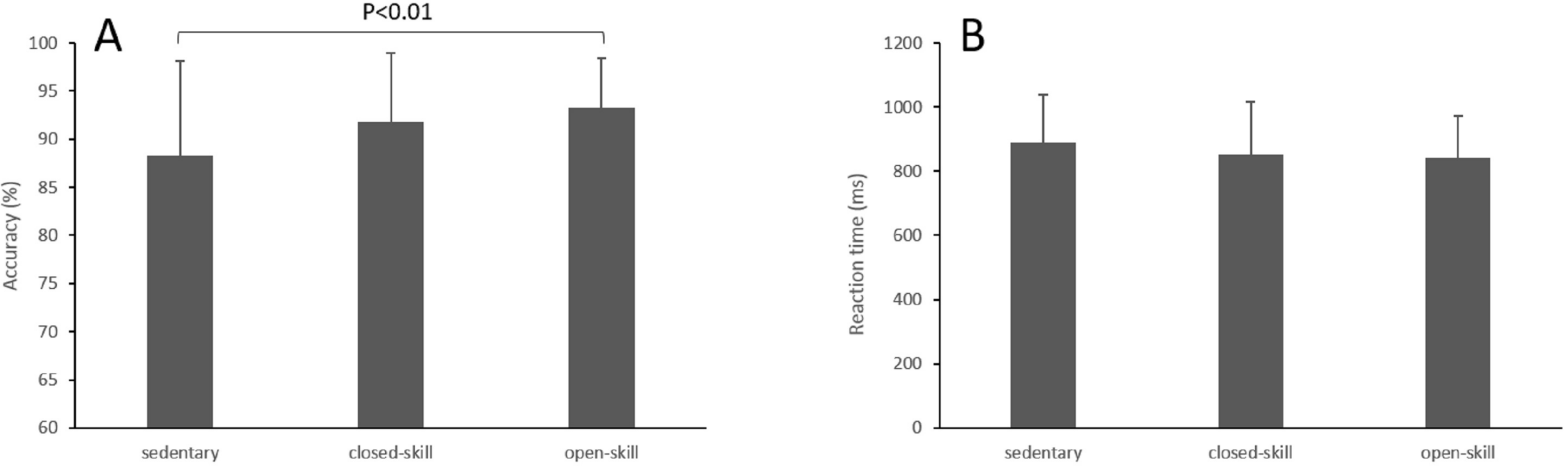
Accuracy (A) and reaction time (B) on visuospatial short-term memory task.

**Figure 5:**
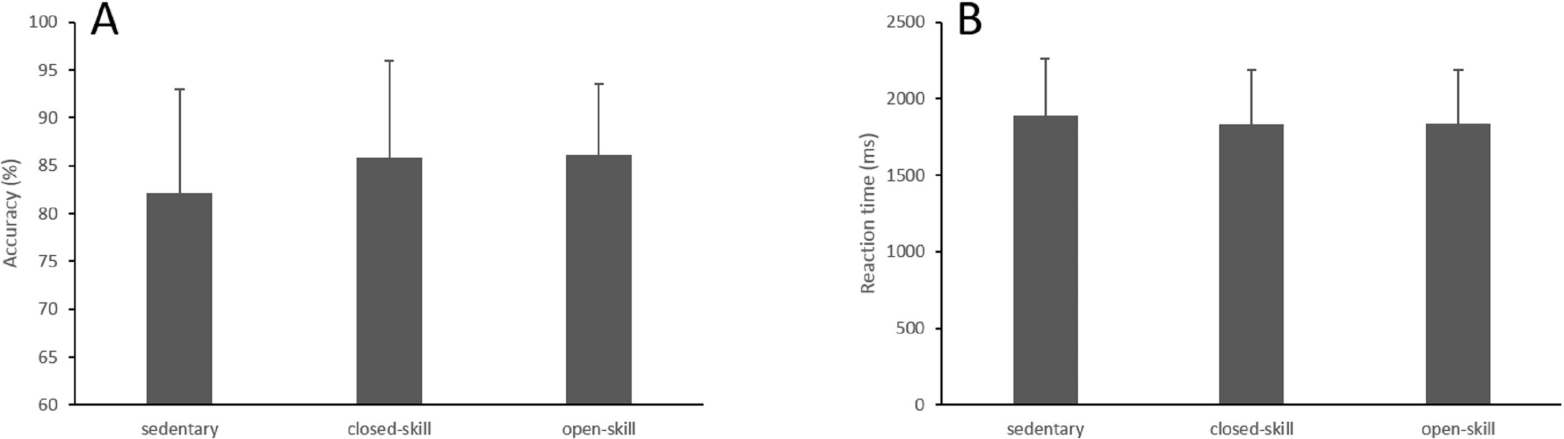
Accuracy (A) and reaction time (B) on visuospatial mental rotation task.

The results showed a significant difference between the groups (F_(2,99)_ = 3.68, *p* = 0.02) on accuracy of VSMT. Post hoc analysis indicated that the open-skill (*p* < 0.01) group reached a higher accuracy than the sedentary group, but there was no difference between the sedentary and closed-skill group, nor between the closed-skill and open-skill group. The reaction time of VSMT was not significantly different among the groups (F_(2,99)_ = 1.05, *p* = 0.36; see Fig. 4). For the VMTT, there were no significant differences on accuracy (F_(2,99)_ = 1.86, *p* = 0.16) or reaction time (F_(2,99)_ = 0.28, *p* = 0.76; see Fig. 5).

## Discussion

The main objective of this study was to investigate the relationship between exercise mode and visuospatial working memory in healthy older adults. Consistent with previous findings (Chang et al., 2013; Erickson et al., 2011), older adults who participated in exercise, regardless of the exercise mode, exhibited better cognitive function than the sedentary older adults. Impairment of working memory is a typical cognitive deficit associated with aging. Working memory has been considered as a significant cognitive factor in theoretical models of cognitive aging (Bizon et al., 2012; Kirova, Bays & Lagalwar, 2015). A previous study reported that physically active older adults exhibited better verbal working memory than the controlled sedentary older adults (Chang et al., 2013). However, there has been a lack of direct evidence to show whether physically active older adults would demonstrate superior visuospatial working memory as well. This was the first study to examine the relationship between exercise mode and visuospatial working memory in older adults to our knowledge. Results of experiment 1 revealed that both the closed-skill group and open-skill group reached a higher accuracy than the sedentary group in the visuospatial working memory task, while no difference between the closed-skill and open-skill groups were found.

The present study also examined the differences of exercise modes on the maintenance and manipulation aspects of visuospatial working memory of older adults. Although experiment 1 did not reveal a difference between the two exercise groups, when visuospatial short-term memory and visuospatial mental rotation were assessed separately in experiment 2, some interesting differences were noticed: open-skill older adults were more accurate than the sedentary older adults in the visuospatial short-term memory task, while no difference was detected between the closed-skill and sedentary older adults. Furthermore, older adults with different exercise modes did not differ in performing the visuospatial mental rotation task.

According to the “continuum” model proposed by Vecchi & Cornoldi (1999), visuospatial working memory is characterized as passive storage or active manipulation depending on the degree of active information processing. Passive storage refers to maintenance of visuospatial information that has not been modified after encoding, while active manipulation refers to the transformation, manipulation, or integration of stored visuospatial information. Active manipulation has shown an earlier onset of aging effects than passive storage in previous studies (Vecchi & Cornoldi, 1999; Vecchi, Richardson & Cavallini, 2005). However, active manipulation did not show more benefit from physical exercise in the present study. The accuracy of each group on visuospatial mental rotation task is much lower than visuospatial short-term memory task, thus it is speculated that aging resulted deterioration of active manipulation was irreversible through either open-skill or closed-skill physical exercises. The present study has also found that open-skill older exercisers perform better than sedentary older adults on the task requiring passive storage processing. The results indicate that passive storage of visuospatial information was more susceptible to open-skill exercise, conforming to the previous studies (Erickson et al., 2011).

The cognitive benefits of open-skill exercises have been reported previously for older adults (Dai et al., 2013; Huang et al., 2014), athletes (Voss et al., 2010; Wang et al., 2013), and even for children with developmental coordination disorder (Tsai, 2009). The cognitive benefits of open-skill exercises may be attributed to the increased cognitive demands in the process of exercise. Previous meta-analyses have confirmed that combining cognitive training with exercise can be effective for improving the cognitive functions of adults with and without cognitive impairment (Law et al., 2014). It has been proposed that exercises performed in a cognitively challenging environment were more effective in inducing neural and cognitive benefits than exercise alone (Fabel et al., 2009). In the present study, open-skill exercisers performed better than closed-skill exercisers and sedentary controls on visuospatial short-term memory. While there were no differences between closed exercisers and sedentary controls, despite the estimated levels of physical activity being significant different between the two groups. The finding was inconsistent with previous findings that better short-term memory ability has been reported in the elderly following aerobic training (Erickson et al., 2009; Erickson et al., 2011). The possible reason for the discrepancy may be the different memory tasks used in the present study. The stimuli used in the present study were the 4 × 4 matrices with four squares of solid black, which were much more complex than the solid dots stimuli used in Erickson’s study (Erickson et al., 2009; Erickson et al., 2011), we speculated that cognitively challenging short-term memory might benefit more from a combination of cognitive training and aerobic exercise.

Some limitations existed in the present study. Firstly, the classification of the participants was based on the questionnaire which was self-reported. Some objective measures such as accelerometers or pedometers should be adopted, maximal oxygen consumption (VO_2_max) could also be measured to indicate the cardiorespiratory fitness in order to control the fitness level across all subjects when examining the relationship between different exercise modes and working memory. Secondly, the cross-sectional design revealed a possible relationship between different exercise modes and visuospatial working memory, but it can hardly conclude a causal relationship. Therefore, intervention studies are needed in the future. Thirdly, the study did not take “screen time” into consideration; it includes watching TV and usage of smart devices, which have been considered a factor mediating cognitive function (Montagni, Guichard & Kurth, 2016; Morita et al., 2016).

## Conclusions

In conclusion, physical exercise is associated with better visuospatial working memory in older adults. Specifically, open-skill exercises which involve high cognitive demands showed selective benefits for maintenance of visuospatial information. The important public health implication is that open-skill exercises may be a more appropriate exercise mode for older adults to maintain or enhance cognitive functions.

##  Supplemental Information

10.7717/peerj.2254/supp-1 Data S1 Raw data

## Additional Information and Declarations

### Data Availability

The following information was supplied regarding data availability: The raw data has been supplied as Data S1.
